# An Ideal Intervention for Cancer-Related Fatigue: Qualitative Findings from Patients, Community Partners, and Healthcare Providers

**DOI:** 10.3390/curroncol31080325

**Published:** 2024-07-30

**Authors:** Nicole Anna Rutkowski, Georden Jones, Jennifer Brunet, Sophie Lebel

**Affiliations:** 1 Faculty of Social Sciences, School of Psychology, University of Ottawa, Ottawa, ON K1N 6N5, Canada; jennifer.brunet@uottawa.ca (J.B.); sophie.lebel@uottawa.ca (S.L.); 2 Mary A. Rackham Institute, University of Michigan, Ann Arbor, MI 48104, USA; gjone046@uottawa.ca; 3 School of Human Kinetics, University of Ottawa, Ottawa, ON K1N 6N5, Canada

**Keywords:** cancer-related fatigue, qualitative, patient-centered care, intervention, implementation, survivorship

## Abstract

Patients consistently rate cancer-related fatigue (CrF) as the most prevalent and debilitating symptom. CrF is an important but often neglected patient concern, partly due to barriers to implementing evidence-based interventions. This study explored what an ideal intervention for CrF would look like from the perspectives of different stakeholders and the barriers to its implementation. Three participant populations were recruited: healthcare providers (HCPs; *n* = 32), community support providers (CSPs; *n* = 14), and cancer patients (*n* = 16). Data were collected via nine focus groups and four semi-structured interviews. Data were coded into themes using content analysis. Two main themes emerged around addressing CrF: “It takes a village” and “This will not be easy”. Participants discussed an intervention for CrF could be anywhere, offered by anyone and everyone, and provided early and frequently throughout the cancer experience and could include peer support, psychoeducation, physical activity, mind–body interventions, and interdisciplinary care. Patients, HCPs, and CSPs described several potential barriers to implementation, including patient barriers (i.e., patient variability, accessibility, online literacy, and overload of information) and systems barriers (i.e., costs, lack of HCP knowledge, system insufficiency, and time). As CrF is a common post-treatment symptom, it is imperative to offer patients adequate support to manage CrF. This study lays the groundwork for the implementation of a patient-centered intervention for CrF in Canada and possibly elsewhere.

## 1. Introduction

Cancer-related fatigue (CrF) has been defined as a “distressing, persistent, subjective sense of tiredness or exhaustion related to cancer or cancer treatment that is not proportional to recent activity and interferes with usual functioning” [1]. Patients diagnosed with various cancers consistently rate fatigue as the most prevalent and debilitating symptom [2]. Approximately 30% of patients living beyond cancer will experience moderate to severe fatigue that persists years after treatment cessation [3,4]. CrF has been found to impact quality of life, contributing to significant socioeconomic losses, disability, isolation, and increased healthcare utilization [5,6].

Due to a growing awareness of CrF and its burden, several organizations have published evidence-based guidelines for its assessment and treatment, including the National Comprehensive Cancer Network (NCCN) [1], Canadian Association for Psychosocial Oncology (CAPO) [7], and more recently, the European Society for Medical Oncology (ESMO) [8]. Guidelines for CrF are relatively consistent and recommend systematic screening for fatigue and interventions including psychoeducation, psychosocial interventions (i.e., cognitive behavioural therapy), physical activity, and supportive expressive therapies. Despite the availability of these guidelines for several years, the dissemination and implementation of evidence-based CrF guidelines continues to be lacking [9,10,11].

In Australia, healthcare providers (HCPs) have reported that the current state of the CAPO guidelines on CrF lacks clinical utility [12]. Presently, the CAPO guidelines contain a plethora of information on possible patient self-management strategies but offer little guidance on how HCPs can support patients in making recommended behavioural changes (e.g., physical activity) [13]. For these reasons, the implementation of these guidelines remains problematic in hospital and community settings, resulting in HCP recommending strategies lacking empirical support (e.g., rest) [14] or recommending evidence-based strategies infrequently [15]. In 2023, a self-management support practice framework was published to provide practical guidance on addressing CrF to address HCPs’ concerns [13]. To increase implementation of treatment strategies for CrF, several strategies have been proposed including leveraging information and communications technologies, prioritizing interventions ready for adaption to local contexts, and creating standardized manuals [16].

The present study aimed to build on findings of a previous study [10] that assessed knowledge of and barriers to the implementation of the CAPO guidelines. In 2021, Jones et al. [10] found professionals’ lack of knowledge and resources along with systemic barriers and breakdowns in communication between patients and providers contributed to the lack of CrF guideline implementation. The purpose of this study was to examine these stakeholders’ perspectives on what an ideal CrF intervention could consist of in the local context using the Knowledge-to-Action (KTA) framework [17,18], a model that aids in knowledge creation and practical application. Owing to its purpose, this study focused on two specific stages of the KTA model: adaptation of knowledge to the local context and identification of barriers. This knowledge will ultimately guide the application of the remaining KTA stages (i.e., selection/tailoring of intervention, monitoring knowledge use, evaluating outcomes, and sustaining knowledge use) in the implementation and evaluation of an evidence-based intervention for CrF in Ottawa, ON, Canada.

## 2. Methods

### 2.1. Participants

This study employed a phenomenological approach [19] in which individuals best positioned to speak on CrF were selected to participate, including patients experiencing CrF, HCPs, and community support providers (CSPs) who worked with patients experiencing CrF. Inclusion criteria for patients, HCPs, and CSPs were: (a) speak English or French; (b) be aged 18–85 years; and (c) be diagnosed with cancer/have experience working with cancer patients. Patients were recruited from hospital and community settings, HCPs were recruited from two local hospitals, and CSPs were recruited via cancer community centres in Ottawa, ON, Canada.

### 2.2. Protocol

This study is a part of a larger mixed methods study that evaluated knowledge of CrF guidelines, assessment, and treatment of CrF in clinical practice [10]. A qualitative research design with focus groups was selected to allow the exploration and understanding of participants’ wants and perceived barriers for implementing an intervention for CrF in Ottawa, ON, Canada. Participants were grouped in separate focus groups. Due to scheduling issues, however, individual interviews with physicians were undertaken. Recruitment and data collection began in July 2016 and was completed in May 2019 once data saturation was reached when analyzing the data for Jones et al.’s study [10]. Research ethics certificates were received from all institutions from which participants were recruited (protocol numbers: #GJ-06-08-15, #H12-15-26 and #20170704-01H). Participants provided written informed consent and completed demographic questionnaires before the focus groups or interviews began. Focus groups were conducted in person and facilitated by pairs of female investigators experienced in psychosocial oncology, a clinical psychologist (S.L.), physical activity researcher (J.B.), and two clinical psychology graduate students (G.J. and N.R.). G.J. conducted three individual interviews by phone and one in person. Focus groups were video/audio-recorded and interviews were audio-recorded only. All audio recordings were transcribed verbatim and imported into NVivo 12 software [20] for analysis. The average focus group length was 90 min with patients and 60 min with HCPs and CSPs. The average interview length was 20 min with HCP-physicians. This study is reported according to standards for reporting qualitative research [21]. The checklist can be found in Supplemental Material.

### 2.3. Measures

Quantitative measures. A brief questionnaire was administered to participants to gather medical, sociodemographic information, and years of experience working in oncology for HCPs and CSPs.

Qualitative interview. Separate semi-structured interview guides were developed to facilitate the patient and professional (HCP, CSP) focus groups and interviews (Supplemental Material). For the present study, questions pertaining to what would constitute an ideal intervention for CrF and anticipated barriers to implementation were explored.

### 2.4. Analyses

#### Qualitative Analyses

To determine what stakeholders believed would be an ideal intervention for CrF, a thematic content analysis was performed by N.R. on the data using NVivo 12 software [20]. N.R. adopted an interpretive phenomenological researcher stance, acting as a bridge between the researchers’ and participants’ horizons of significance. Her reflexive stance was guided by her experience as a cancer survivor and her clinical and research training in psychosocial oncology [22,23]. Adhering to Braun and Clarke’s methods [24], inductive data analysis began by becoming acquainted with the data through a first reading of all transcripts, followed by generating codes for pertinent features of the data that aligned with the current study. After this, themes and subthemes were generated from existing codes, and each theme was reviewed to ensure information coded was appropriate for the theme. Then, subthemes and themes were defined and labeled. Last, exemplar quotes were located to provide support for conclusions. Triangulation was performed to look for similarities and differences between patients’, HCPs’, and CSPs’ viewpoints within themes [25]. Thirty percent of the focus groups were double coded by a second coder. Themes were discussed until consensus was reached.

## 3. Results

### 3.1. Participants

A total of 62 participants were recruited—16 patients, 32 HCPs, and 14 CSPs. The 16 patients were divided into three focus groups (two in English [*n* = 5 and *n* = 4], one in French [*n* = 7]). Focus groups took place at the Ottawa Hospital and Montfort hospital, refreshments were provided, and cost of parking was reimbursed. Four HCP focus groups were conducted—one with members of an interdisciplinary breast cancer team (French *n* = 4), one with members of an interdisciplinary psychosocial oncology team (*n* = 8), one group of oncology nurses (*n* = 10), and one group of radiation technicians (*n* = 10), at their respected hospitals of employment. Three individual interviews were conducted with HCP-physicians over the telephone and one in person. Two CSP focus groups (*n* = 7 and *n* = 7) were conducted and hosted at two community cancer support centers in Ottawa, ON, Canada. See Table 1 for an overview of the patients’, HCPs’, and CSPs’ characteristics.

### 3.2. Themes

Two overarching themes emerged from the data around addressing CrF: “It takes a village” and “This will not be easy”. For the first theme, participants discussed provider, timing, and location of the intervention (anywhere, offered by anyone and everyone, and provided early and frequently through the cancer experience) and the components of an intervention they wanted (education, physical activity, mind–body, interdisciplinary care, and co-ordination of care). For the second theme, patients, HCPs, and CSPs described several potential barriers to implementation, including patient barriers (i.e., patient variability, accessibility, online literacy, and overload of information) and systems barriers (i.e., costs, lack of HCP knowledge, system insufficiency, and time).

#### 3.2.1. Theme 1: “It Takes a Village”

##### Provider, Timing, and Location of the Intervention

Participants described the program could be located anywhere so long as it was accessible, well located, welcoming, and provided centralized services. In the words of a patient: “*I don’t care where I go. It doesn’t matter to me as long as [the programming] is good when I get there*” and an HCP stated, “*I think it’s really important that these [services] are offered closer to home*”.

A subtheme of anyone and everyone early and frequently emerged where participants described that people from many disciplines or peers could provide education on fatigue and emphasized the importance of it being someone who has first contact or regular contact with the patient. An HCP described: “*I think the first person that sees them should give them something because even by the time they can see their social worker it’s often too late. It needs to be caught before it happens*”, and a CSP added, “*I think it would be a multitude of different people that could speak to the different aspects [of CrF]*”. Participants discussed the importance of educating patients about fatigue early on and frequently, due to difficulty processing information at diagnosis. A CSP described discussing fatigue: “*I think the earlier the better…early and often…knowing why [fatigue] is there always makes you cope with things better*”; and in the words of a patient: “*But for me it’s more important to know ahead of time and not knowing that I would have the fatigue I did not prepare for it. And I would have done things differently…*”.

##### Desired Components of an Intervention

Participants spoke to the importance of peer support, not feeling so alone, and normalizing the experience of fatigue. In the words of an HCP: “*If they could talk about fatigue, normalize it, and it’s to be expected and if people share their challenges about going back to work…*” and a patient added: “*There should be one afternoon a week where everyone could meet and talk about the fatigue. Whether it’s an exercise session…or 20 min of socializing. Because you won’t feel as isolated*”.

Participants recommended educating patients as well as HCPs on CrF. In the words of a CSP and patient, respectively: “*When I think of unmet needs for clients, I think a huge one is education around CrF. And knowing “What can I do?*” and “*Maybe if someone sat down and talked about the fatigue, because no one really talked to me about it*”. Participants expressed wanting information on the connection between fatigue and other symptoms such as mood, strategies to manage fatigue, information that CrF is common prior to treatment, and recommendations of strategies to manage and communicate about CrF.

Patients, HCPs, and CSPs discussed in-person supervised exercise sessions; believing these would enhance patients’ motivation for physical activity and reduce isolation through contact and support from others experiencing CrF (if group-based). In the words of one patient: “*Kind of a support group, but not with the structure where everybody sits down in a circle and talks. I want it to be dynamic, you need to be pushed a little bit to exercise*”. Participants suggested yoga, walking, or running groups and offered that depending on the activity, these could be led by peers to increase sustainability by reducing costs. Additionally, cognitive behavioural therapy elements were raised by CSPs such as identifying and addressing individual barriers to physical activity. A CSP explained: “*For somebody to say, “you should walk 30 min” that’s fine but the next question is well “why aren’t you doing that?” So, what is that barrier? Is it because you think you don’t have time? Okay well let’s talk about that*”.

Participants also raised mind–body interventions, such as mindfulness, meditation, art therapy, and stress reduction. CSPs expressed the use of mindfulness as a facilitator of behavioural change through patient empowerment, increased agency, and acceptance. A CSP explained: “*For a lot of people, a mindfulness-based approach as a part of intervention–that is very empowering and helpful. I mean not only is the whole piece of being in the moment and meditating energy-giving, it also engenders more self-awareness*”.

Participants described the need for interdisciplinary care and patient-centric integrative approach, wherein an intervention addresses the multifactorial nature of CrF through an interdisciplinary team. A patient described: “*I think first off it should be integrative. Here is the patient, here is your medical team, here is your psychosocial oncology contact, here is your physician, here is your nutritionist, here is your radiation-oncologist. And we are your team*”. An HCP added: “*In a perfect world, we go back to having a group where we have an [Occupational Therapist] and a [Physio Therapist] and a dietician and maybe a social worker or a psychologist involved in counselling*”.

Participants discussed a desire for coordination of care, describing a lack of integration of services and a need for greater communication between hospitals, community partners, and patients, particularly around what services are available for patients. Patients and HCPs expressed that ideally, there would be someone following patients who could provide regular check-ups and guidance. A patient described: “*Someone who can get in touch with you. Like that call that I got from [the hospital] really gave me a lift of “Wow, they didn’t just forget about me!” It was three months later and “How are you doing? Do you need anything? Is there a service that you’re looking for?*”.

#### 3.2.2. Theme 2: “This Will Not Be Easy”

##### Patient Barriers

The accessibility subtheme captured barriers to offering an intervention that would be accessible for all (e.g., rural communities, individuals with lower socioeconomic status). Participants identified barriers such as difficulties with attending an intervention in person due to distance, financial restraints (i.e., cost of parking or interventions/services), and symptom interference. A CSP suggested: “*Offering [the intervention] somewhere that is accessible—like free parking and all that*”. A cancer patient further explained how post-treatment symptoms interfered with in-person programs, “*I live in the middle of nowhere…but I think part of my brain fog is also time management and recognizing how long things take, getting ready and out the door on time is really difficult for me*”.

Online literacy subtheme captured concerns that patients may find online interventions challenging due to potential limited online literacy. As one patient reported: “*Being of different age than 90% of the people in here, I’m not comfortable with the internet*”.

The subtheme of information overload described how HCPs and CSPs felt that patients were overwhelmed and overloaded with information during their cancer trajectory, which made it challenging for them to take in new information. As one HCP shared, “*it can be like a deer in the headlights. I’ve had patients actually say sometimes it’s like support overload… you need this, you need to do this, how about you go here?*”

Participants also raised concerns over patient variability that captured differences between patients regarding motivation, willingness to implement change, self-efficacy, level of family support, education level, language barriers, and personality (i.e., shyness) that could impact their ability to engage in a CrF intervention successfully. An HCP explained: “*Sometimes it’s their mentality. They feel like they just have to go home and deal with [the fatigue], they don’t really think there’s a way to try to solve the issue*”, while a CSP added that patients are “*not necessarily used to in North America taking control of [their] own health and having to do things, [they] may have to change [their] diet or might have to exercise, these things aren’t easy to do…there’s no pill to fix CrF*”.

### 3.3. Systems Barriers

There were several systems barriers raised to implementing an intervention for CrF, including cost, lack of knowledge, system inefficiencies, and time constraints. Participants expressed concerns related to hospital administration, funding, and procedures that made it difficult to get referrals for patients or justify services that may be deemed “non-essential” such as for CrF. Participants also expressed difficulty with collaboration and having patients referred to different programming. See Table 2 for additional illustrative quotes.

## 4. Discussion

This study summarized the perspectives of stakeholders on what an ideal CrF intervention could consist of in the local context in Ottawa, ON, Canada. The two themes that emerged from the data (“It takes a village” and “This will not be easy”) capture the recognized importance and impact of fatigue while also acknowledging there are many personal and systemic barriers that make implementing CrF interventions challenging. Participants expressed a CrF intervention could be facilitated by anyone and everyone, housed anywhere, and offered early and frequently through the cancer experience, which may speak to the level of need for CrF programming and resources in the local context. Peer support was discussed as an important component of CrF programming. Several interventions for CrF were raised including education, physical activity, mind–body, interdisciplinary care, and coordination of care. The perceptions of HCPs, CSPs, and patients may help direct much-needed efforts in implementing programming and resources for CrF in Ottawa and elsewhere.

Based on insights from our larger study [10] and in accordance with other studies [26], CrF guideline implementation continues to be lacking due to a lack of HCP knowledge around CrF and a breakdown in patient–provider communication, leading to patients being dissatisfied with care for CrF. In our study, participants indicated that any HCP could facilitate CrF management and that an intervention should be offered early and frequently across the cancer experience. The CAPO guidelines do recommend early and thorough evaluation of fatigue, education on CrF for all patients, and continued follow-up, yet this poses a significant challenge due to the lack of education, clinically useful resources, and training opportunities for HCPs around CrF [26]. Indeed, a recent study found that 87% of HCPs rated having limited or moderate expertise with CrF despite years of practice [26]. It may therefore be paramount to prioritize HCPs’ education on CrF. Another consideration is the lack of leadership around fatigue which may be resulting in a diffusion of responsibility among HCPs [27], as well as a lack of prioritization at organizational and policy levels [26].

Participants expressed an intervention for CrF could be offered anywhere (i.e., community or hospital) so long as it was accessible. However, patients experience many barriers to face-to-face programming as detailed in this study and reported in the literature with a long commute time being the most commonly cited barrier [25], as well as experiencing fatigue [26], which warrant consideration. A virtual CrF intervention may be best positioned to reduce significant barriers like accessibility, costs, informational overload, and time constraints but may exclude patients who feel uncomfortable with technology. Additionally, eHealth interventions may offer more flexibility for components such as intensity and duration of an intervention and be better personalized to patient variability and information presented, thereby being more likely to maintain patient motivation, satisfaction, and adherence [28]. Indeed, in 2022, Beenhakker et al. [28] published an overview of 35 existing CrF interventions and the variations in patient preferences among these interventions, which may help guide patients or practitioners in selecting an appropriate eHealth program. Further, based participants’ perceptions, programming with peer support should be offered when possible as it appears to contribute to a sense of normalcy and connection. Social support has also been found to facilitate physical activity motivation, an important evidence-based recommendation for CrF [29]. In-person exercise sessions may be a component of interdisciplinary care and offer inherent social support.

Importantly, participants expressed interest in education around CrF which may indicate an unmet informational need around CrF. Indeed, a recent study found that only 23% of patients were informed of treatment options for CrF [30]. Further, most patients in the study indicated they needed to initiate the conservation around fatigue with HCPs [30] as opposed to HCPs inquiring about CrF. This may speak to the lack of dissemination of various resources that have been available for several years, including published educational booklets by Cancer Care Ontario in 2016 [31], Alberta Health services in 2017 [32], and Eastern Health in 2018 [33]. Many of these resources also contain video education for patients, as well as other international resources [34,35,36]. Given that existing resources and evidence-based treatments have been created, organizations may wish to focus on adaptation and practitioner education around available resources for CrF as a cost-effective step to begin to address this unmet information need. A virtual psychoeducation intervention on the management of CrF may be a beneficial and cost-effective approach; psychoeducation interventions have shown to significantly improve the management of CrF among people living beyond cancer [37], especially when offered in a group setting and incorporating cognitive behavioural therapy [38].

Participants raised the benefits of a case coordinator. Navigation services have been found to improve adherence to surveillance appointments, decision making, satisfaction with care, and quality of life; however, limited evidence has been found that they improve CrF [39]. Several systems barriers were noted in our study that have been found by other studies [12,26]. It is evident that to increase the implementation of CrF guidelines, significant policy changes are needed, including increased funding, protected education time for HCPs, improved care pathways, and prioritization of CrF within organizations.

## 5. Limitations

The data were collected prior to the COVID-19 pandemic, which may have altered patients’, HCPs’, and CSPs’ perceptions on what may be an ideal intervention especially regarding virtual or in-person settings. Further, the data were collected in Ottawa, ON, Canada from participants who are Caucasian/White from higher socioeconomical backgrounds and well-educated. This may limit the transferability of the results.

## 6. Conclusions and Future Directions

This study emphasizes the critical need for CrF programming and implementation of evidence-based treatment. Future studies should prioritize effectiveness-implementation studies that accelerate the development of clinically useful resources and are adapted to local settings. Virtual CrF interventions may be best positioned to reduce barriers to accessing critical support to managing CrF, and organizations may wish to prioritize education and the adaptation of existing CrF resources and programs. Lastly, policy changes are needed to address significant systems barriers like costs, system inefficiencies, and time constraints.

## Figures and Tables

**Table 1:** Sociodemographic information [10].

Patients		
	Mean (M)	SD
Age	55.56	10.30
Time since diagnosis (years)	5.31	2.55
Time since treatment completion (years)	3.13	1.92
	N (%)	
Gender		
Man	7 (43.8%)	
Women	9 (56.3%)	
Language		
English	6 (37.5%)	
French	10 (62.5%)	
Employment		
Full-time	3 (18.8%)	
Part-time	3 (18.8%)	
No	10 (62.5%)	
Born in Canada		
Yes	16 (100%)	
Ethnicity		
Caucasian/White	16 (100%)	
Diagnosis		
Prostate	2 (12.5%)	
Breast	8 (50%)	
Colorectal	1 (6.3%)	
Stomach	1 (6.3%)	
Multiple	3 (18.8%)	
Colon	1 (6.3%)	
Chemotherapy		
Yes	8 (50%)	
Radiation		
Yes	10 (62.5%)	
Surgery		
Yes	10 (62.5%)	
Income (CAD $)		
<40,000	4 (25%)	
40,000–59,999	2 (12.5%)	
60,00–79,999	1 (6.3%)	
80,000–99,999	1 (6.3%)	
100,000–150,000	6 (37.5%)	
>150,000	1 (6.3%)	
Do not know	1 (6.3%)	
Healthcare Providers		
	M	SD
Age	42.06	9.75
Work experience (years)	11.05	8.21
	N (%)	
Gender		
Man	5 (15.6%)	
Women	27 (84.4%)	
Title		
Radiation technician	10 (31.3%)	
Registered nurse	10 (31.3%)	
Social worker	4 (12.5%)	
Psychologist	2 (6.3%)	
Radiation oncologist	2 (6.3%)	
Dietician	1 (3.1%)	
Surgeon	1 (3.1%)	
Palliative care physician	1 (3.1%)	
Physiotherapist	1 (3.1%)	
Community Support Providers		
	M	SD
Age	43.86	11.20
Work experience (years)	3.93	3.13
	N (%)	
Gender		
Man	2 (14.3%)	
Women	12 (85.7%)	
Title		
Cancer coach	7 (50%)	
Hypnotherapist	1 (7.1%)	
Natural Chinese medicine	1 (7.1%)	
Registered massage therapist	1 (7.1%)	
Naturopathic doctor	1 (7.1%)	
Yoga therapist	1 (7.1%)	
Manager	1 (7.1%)	
Research fellow	1 (7.1%)	

**Table 2:** Qualitative excerpts from patients, community support providers, and healthcare providers.

Theme	Subtheme	Quote
**Provider, Timing, and Location of the Intervention**	Anywhere	“*It’s not one or the other [referring to community or hospital] …I think it could be in any location, but I think it’s that education piece…”—CSP* “*Accessible, for everybody.*”*—Patient*
	Anyone and everyone, early and frequently	“*I think if it was something that was part of their regular medical practice.*”*—CSP* “*I think the doctors and everybody that we need to see in the medical field. They need to be more aware of how fatigue affects us. I think they take it too lightly… it’s a ball floating and no one really grabs it.*”—Patient
**Desired Components of an Intervention**	Peer support	“*Maybe there’s an opportunity to have like a peer-to-peer advocacy group, [for] people who have lived with fatigue, and are living with it…*”—Patient “*If I had talked to other people dealing with the fatigue maybe I wouldn’t be so anxious and stressed and feeling like what’s happening to me…*”—Patient
	Education on CrF	“*I think that intervention or education as we’ve brought up is a huge piece of reassurance around what they’re coping with, and then to have the intervention—it should be holistic, it should talk about all the different things they can do, you know exercise, nutrition. How stress, how brain fog, how all these things impact their energy levels and what they can do about that.”—CSP*
	Physical activity	“*If we could offer like an exercise class, that’s very basic, you could get a bunch of people and do it together.*”—HCP
	Mind–body	“*The neat thing about mindfulness is that I think it’s a strategy that could be multi-modal. Like it could cover other aspects of difficulty with cancer too. But I think in terms of fatigue it is a useful intervention.*”–CSP
	Interdisciplinary care	“*From my point of view, I just want to mention too that having a little bit of a better interdisciplinary team.*”—HCP
	Coordination of care	“*A resource person, capable of, as I said earlier, sensitivity, being able to situate, being able to listen...*”—Patient
**Patient Barriers**	Accessibility	“*I find that there’s a lot of resources but there’s no follow through to get to them…especially a patient coming from a rural setting.*”—HCP
	Online literacy	“*A lot of them are older. So webinars might be a challenge for them.”—CSP* “*…those of us that are older and our patients are even older, we can’t just google the problem and solve it or go to a YouTube video.*”—HCP
	Information overload	“*I personally try to tailor my techniques and everything to their education level like use simpler words, shorter sentences,* etc. *But sometimes it’s just too much for them, and then you give them a piece of paper and then it’s just too much and overwhelming.*”—HCP
	Patient variability	“*Because we can recommend everything until we’re blue in the face but if the patient doesn’t want to do it... or if they’re not interested or if it’s not what they like…*”—HCP “*There’ll be people who are perhaps shy and don’t want to work in a group and will individually go online.*”—CSP
**Systems Barriers**	Cost	“*I understand it from a financial perspective, we just can’t keep everything here at the hospital and we tell the patient what they can do. And we hope that they do it. But in some cases, they won’t unless they’re being guided to do it.*”—HCP “*I just worry that in a hospital system an [intervention for CrF] will be seen as something that is non-essential and will get moved out very quickly*.”—HCP “*There’s what 300 cancer charities, we’re all fighting over donor dollars…”—CSP*
	Lack of knowledge	“*Fatigue was such a profound symptom for patients later on. So, I think health care provider education or lack of is a barrier.*”—HCP
	System inefficiencies	“*We have to refer patients for many things, and I know sometimes people say that process isn’t complicated. But when there are 10 or 20 things to refer for, the simpler it is the more likely we are to think about it and use it.*”—HCP “*We work really hard to partner but there is a challenge in that…we know oncologists say to their patients, they’re not allowed to refer directly to any specific organization.*”—CSP
	Time constraints	“*Well, not just that I think it’s more time to figure things out. It’s time with a person. You know, we don’t have time…*”—HCP

## Data Availability

The data presented in this study are available on request from the corresponding author due to privacy or ethical restrictions.

## Supplementary Materials

The following supporting information can be downloaded at: https://www.mdpi.com/article/10.3390/curroncol31080325/s1, Supplementary File: Standards for Reporting Qualitative Research (SRQR). Patient Interview Guide. HCP and CSP Interview Guide. Reference [21] is cited in the supplementary materials.
