# Supplementary material for: An Ideal Intervention for Cancer-Related Fatigue: Qualitative Findings from Patients, Community Partners, and Healthcare Providers
Source: Curr Oncol. 2024 Jul 30;31(8):4357–68. doi: 10.3390/curroncol31080325 (PMC11353202; doi:10.3390/curroncol31080325)
Supplement: Supplementary file 1 [file curroncol-31-00325-s001.zip › curroncol-3112731-supplementary.pdf]

Standards for Reporting Qualitative Research (SRQR)*

http://www.equator-network.org/reporting-guidelines/srqr/

Title and abstract

	Page/line no(s).
**Title** - Concise description of the nature and topic of the study Identifying the study as qualitative or indicating the approach (e.g., ethnography, grounded theory) or data collection methods (e.g., interview, focus group) is recommended	Pg. 1
**Abstract** - Summary of key elements of the study using the abstract format of the intended publication; typically includes background, purpose, methods, results, and conclusions	Pg. 2

Introduction

**Problem formulation** - Description and significance of the problem/phenomenon studied; review of relevant theory and empirical work; problem statement	Pg. 3; line: 58-78
**Purpose or research question** - Purpose of the study and specific objectives or questions	Pg. 3; line:80-91

Methods

**Qualitative approach and research paradigm** - Qualitative approach (e.g., ethnography, grounded theory, case study, phenomenology, narrative research) and guiding theory if appropriate; identifying the research paradigm (e.g., postpositivist, constructivist/ interpretivist) is also recommended; rationale**	Pg. 4; line 96-98
**Researcher characteristics and reflexivity** - Researchers' characteristics that may influence the research, including personal attributes, qualifications/experience, relationship with participants, assumptions, and/or presuppositions; potential or actual interaction between researchers' characteristics and the research questions, approach, methods, results, and/or transferability	Pg. 4; line: 115-118, 137
**Context** - Setting/site and salient contextual factors; rationale**	Pg. 4; lines 105-122
**Sampling strategy** - How and why research participants, documents, or events were selected; criteria for deciding when no further sampling was necessary (e.g., sampling saturation); rationale**	Pg. 4; line: 96-98; 110-112
**Ethical issues pertaining to human subjects** - Documentation of approval by an appropriate ethics review board and participant consent, or explanation for lack thereof; other confidentiality and data security issues	Pg. 4; line: 112-113
**Data collection methods** - Types of data collected; details of data collection procedures including (as appropriate) start and stop dates of data collection and analysis, iterative process, triangulation of sources/methods, and modification of procedures in response to evolving study findings; rationale**	Pg. 4; Line: 110-113; 115-122

**Data collection instruments and technologies** - Description of instruments (e.g., interview guides, questionnaires) and devices (e.g., audio recorders) used for data collection; if/how the instrument(s) changed over the course of the study	Pg 4; line 125-132
**Units of study** - Number and relevant characteristics of participants, documents, or events included in the study; level of participation (could be reported in results)	Pg. 5; line 149-159, Pg 13. Table 1
**Data processing** - Methods for processing data prior to and during analysis, including transcription, data entry, data management and security, verification of data integrity, data coding, and anonymization/de-identification of excerpts	Pg 4; line 119-120
**Data analysis** - Process by which inferences, themes, etc., were identified and developed, including the researchers involved in data analysis; usually references a specific paradigm or approach; rationale**	Pg. 4; line; 136-146
**Techniques to enhance trustworthiness** - Techniques to enhance trustworthiness and credibility of data analysis (e.g., member checking, audit trail, triangulation); rationale**	Pg 5; line 143-146

Results/findings

**Synthesis and interpretation** - Main findings (e.g., interpretations, inferences, and themes); might include development of a theory or model, or integration with prior research or theory	Pg 5-7; lines 169-272
**Links to empirical data** - Evidence (e.g., quotes, field notes, text excerpts, photographs) to substantiate analytic findings	Pg 5-7; lines 169-272; pg. 15-16; Table 2

Discussion

**Integration with prior work, implications, transferability, and contribution(s) to the field -** Short summary of main findings; explanation of how findings and conclusions connect to, support, elaborate on, or challenge conclusions of earlier scholarship; discussion of scope of application/generalizability; identification of unique contribution(s) to scholarship in a discipline or field	Pg. 8; lines 274-333
**Limitations** - Trustworthiness and limitations of findings	Pg. 9; lines 335-340

Other

**Conflicts of interest** - Potential sources of influence or perceived influence on study conduct and conclusions; how these were managed	Pg 1; line 28
**Funding** - Sources of funding and other support; role of funders in data collection, interpretation, and reporting	Pg. 1; lines: 25-27

*The authors created the SRQR by searching the literature to identify guidelines, reporting standards, and critical appraisal criteria for qualitative research; reviewing the reference lists of retrieved sources; and contacting experts to gain feedback. The SRQR aims to improve the transparency of all aspects of qualitative research by providing clear standards for reporting qualitative research.

**The rationale should briefly discuss the justification for choosing that theory, approach, method, or technique rather than other options available, the assumptions and limitations implicit in those choices, and how those choices influence study conclusions and transferability. As appropriate, the rationale for several items might be discussed together.

Reference:
O'Brien BC, Harris IB, Beckman TJ, Reed DA, Cook DA. **Standards for reporting qualitative research: a synthesis of recommendations.** *Academic Medicine*, Vol. 89, No. 9 / Sept 2014
DOI: 10.1097/ACM.0000000000000388

Patient Interview Guide

Opening word:

- The group leaders will introduce themselves and will present the objective of the focus group: To explore patients' experiences with cancer-related fatigue (CRF)
- The group leaders will ensure that all participants still consent to participate in the study and to being voice-recorded and to be filmed
- Group leaders will explain that they are responsible for the confidentiality and the anonymity of the participants and will ask the participants to ensure the confidentiality of the others in the group

CRF questions for patients:

1. Can you briefly describe your experience with CRF?

2. Can you explain what motivated you to report or withhold your CRF from any or all of your health care providers?

3. Has your health care provider assessed your CRF?

    • Probe: How did they do this?
    • Probe: Did they ask you questions about your CRF? Like what?
    • Probe: Did they do blood tests? Do you recall discussing the results of these blood tests?
    • When did this happen?

4. Have you ever received information to manage your CRF?

    • Probe: Who provided you with this information?
    • Probe: When did they do this?
    • Probe: How helpful was this to you?

5. Have you ever received strategies to manage your CRF?

    • Probe: Who provided you with these strategies?
    • Probe: When did they do this?
    • Probe: How helpful was this to you?

6. How and when would you have liked information and strategies to be given to you?

7. What do you do to manage your CRF?

8. What would an ideal approach to help manage your CRF look like?

- Probe: We know that some things like physical activity, energy conservation (i.e., pacing yourself with periods of relaxation between periods of activity) as well as psychotherapy that can help you change unhelpful thoughts about feeling fatigued are useful to help manage CRF. Would you see yourself doing any of these things?

9. What might get in the way of continuing with the recommendations for the management of CRF after the treatment ends?

- Probe: What might make it easier?

10. Who do you think should be responsible for helping your manage your CRF? Where should you receive assistance to manage your CRF? And at what point in the cancer journey would you have liked to first receive assistance?

11. Is there anything you would like to add about CRF and your experience with it?

HCP and CSP Interview Guide

Opening word:

- The group leaders will present themselves and will present the objective of the focus group
- The group leaders will ensure that all participants still consent to participating in the study and to being filmed
- Group leaders will explain that they are responsible for the confidentiality and the anonymity of the participants and will ask the participants to ensure the confidentiality of the others in the group

CRF questions for HCPs:

1. What is your understanding of the components of CRF or how would you define CRF?
    - What is your understanding of the impact of CRF?
2. In your experience, how often and at what point do patients report CRF?
    - Probe: When do you feel CRF is more prevalent?
3. How often do you actively assess patients for CRF in your practice and how do you do this?
    - *Probe: Do you ask questions? Do you run specific tests? Which ones?*
4. What do you want to rule out in patients with CRF before you recommend self-care strategies?
5. What treatment(s) for CRF do you recommend to your patients?
    - *Probe: Why do you recommend these? What informs your recommendations?*
    - *Probe: Do you vary your recommendations based on the patient's trajectory in their cancer journey (i.e. active treatment, end of life, survivorship)? What do you recommend for patients newly diagnosed with cancer? What about those on treatment? What about for pts who have just completed tx? And finally what do you suggest in patients who are in well-follow-up/survivorship?*
    - *Probe: Do you recommend physical activity, CBT, energy conservation strategies?*
6. Can you describe your level of familiarity with the CAPO/NCCN/CCO/COSTAR guidelines on CRF?
7. If we wanted to improve the way we manage CRF, what would be an ideal assessment and intervention plan?
    - *Probe: Who do you think should provide patients with this assistance?*
    - *Probe: Where should patients receive this assistance?*
    - *Probe: How should patients receive this assistance? (e.g., face-to-face, online)*
8. What barriers do you experience in practice for the assessment and intervention for CRF? What barriers to you think patients experience? How could they be avoided?

9. What could be done to ensure the assessment and management of CRF is sustainably done in the future?

10. Do you have any further comments or suggestions about CRF?
